# Theoretical perspectives on central chemosensitivity: CO_2_/H^+^-sensitive neurons in the locus coeruleus

**DOI:** 10.1371/journal.pcbi.1005853

**Published:** 2017-12-21

**Authors:** Maria C. Quintero, Robert W. Putnam, Juan M. Cordovez

**Affiliations:** 1 Biomedical Engineering Department, Universidad de Los Andes, Bogotá, Colombia; 2 Department of Neuroscience, Cell Biology, and Physiology, Boonshoft School of Medicine, Wright State University, Dayton, Ohio, United States of America; Northeastern University, UNITED STATES

## Abstract

Central chemoreceptors are highly sensitive neurons that respond to changes in pH and CO_2_ levels. An increase in CO_2_/H^+^ typically reflects a rise in the firing rate of these neurons, which stimulates an increase in ventilation. Here, we present an ionic current model that reproduces the basic electrophysiological activity of individual CO_2_/H^+^-sensitive neurons from the locus coeruleus (LC). We used this model to explore chemoreceptor discharge patterns in response to electrical and chemical stimuli. The modeled neurons showed both stimulus-evoked activity and spontaneous activity under physiological parameters. Neuronal responses to electrical and chemical stimulation showed specific firing patterns of spike frequency adaptation, postinhibitory rebound, and post-stimulation recovery. Conversely, the response to chemical stimulation alone (based on physiological CO_2_/H^+^ changes), in the absence of external depolarizing stimulation, showed no signs of postinhibitory rebound or post-stimulation recovery, and no depolarizing sag. A sensitivity analysis for the firing-rate response to the different stimuli revealed that the contribution of an applied stimulus current exceeded that of the chemical signals. The firing-rate response increased indefinitely with injected depolarizing current, but reached saturation with chemical stimuli. Our computational model reproduced the regular pacemaker-like spiking pattern, action potential shape, and most of the membrane properties that characterize CO_2_/H^+^-sensitive neurons from the locus coeruleus. This validates the model and highlights its potential as a tool for studying the cellular mechanisms underlying the altered central chemosensitivity present in a variety of disorders such as sudden infant death syndrome, depression, and anxiety. In addition, the model results suggest that small external electrical signals play a greater role in determining the chemosensitive response to changes in CO_2_/H^+^ than previously thought. This highlights the importance of considering electrical synaptic transmission in studies of intrinsic chemosensitivity.

## Author summary

The sensory mechanism by which changes in CO_2_ and H^+^ levels are detected in the brain is known as central chemoreception. Altered chemoreception is common to a wide variety of clinical conditions, including sleep apnea, sudden infant death syndrome, hyperventilation, depression, anxiety and asthma. In addition, CO_2_/H^+^-sensitive neurons are present in some regions of the brain that have been identified as drug targets for the treatment of anxiety and panic disorders. We are interested in understanding the cellular mechanisms that determine and modulate the behavior of these neurons. We previously investigated possible mechanisms underlying their behavior in rats to elucidate whether they respond to changes in intracellular or extracellular pH, CO_2_, or a combination of these stimuli. To study the roles that signals and ion channel targets play in individual neurons we develop mathematical models that simulate their electrochemical behavior and their responses to hypercapnic and/or acidotic stimuli. Nowadays, we are focused on using computational tools to explore the firing pattern of such neurons in response to chemical (CO_2_/H^+^) and electrical (synaptic) stimulation. Our results reveal significant effects of electrical stimulation on the responses of brainstem neurons and highlight the importance of considering synaptic transmission in experimental studies of chemosensitivity.

## Introduction

Central chemoreception is a neuronal sensory mechanism by which changes in CO_2_ and H^+^ levels in the brain are detected [1–3]. It occurs in specialized CO_2_/H^+^-sensitive centers in the brainstem that are involved in the neuronal network that regulates autonomic ventilation [4–10]. Regular ventilatory movements are controlled by respiratory neurons in the brainstem, which generate an appropriate respiratory rhythm and control the motor neurons that innervate the respiratory muscles [11–14]. Even small alterations in CO_2_/H^+^ levels in the blood and/or cerebrospinal fluid cause changes in ventilation. Brainstem neurons are considered the main sensory elements in the homeostatic regulation of respiratory gases [15,16], and when these neurons are exposed to elevated CO_2_/H^+^ (hypercapnia and/or acidosis), there is a noticeable increase in their firing rate. This change in firing rate can be triggered by several signaling pathways alone or in combination, such as a decrease in intracellular or external pH [17,18], an increase in intracellular HCO_3_^−^ [19] and/or a direct increase in CO_2_ [20].

The changes in firing rate of neurons from chemosensitive regions have been investigated under conditions of hypercapnic acidosis (HA) in some areas of the brainstem, such as the retrotrapezoid nucleus [21], nucleus tractus solitarii [22,23], locus coeruleus [24–26] and the medullary *raphe* [27]. It is often assumed that these neurons are intrinsically responsive to changes of CO_2_, which means that they not simply respond to altered synaptic input from other chemosensitive neurons. In particular, neurons from LC have been demonstrated to be responsive when exposed to altered levels of CO_2_ in the presence of synaptic block media. However, it remains unclear whether their firing-rate response to increased levels of CO_2_/H^+^ can be completely attributed to intrinsic mechanisms of individual neurons or if the response is mediated in part by unnoticed inputs from chemical synapses or gap junctions [18,23,28].

Moreover, since there have been postulated a growing number of signals implicated in the chemosensitive response of individual neurons from the LC region [17–20], the knowledge about the various signals, both excitatory and inhibitory, is getting so complex that our ability to understand the interactions between these differing components needs to be aided by mathematical and computational approaches. In particular, a single-cell neuron model is required to elucidate the effect of individual signals and their interaction on the chemosensitive response of individual neurons. Such an approach would help to elucidate whether their spike patterns and discharge frequencies are significantly affected by individual signals, by the combined effect of different stimuli, or by a contribution of small external inputs.

Experimental evidence suggests that neurons from different chemosensitive regions are similar in their basal discharge frequencies and spiking patterns in response to current injection [27–29]. On the other hand, some mathematical models of the Hodgkin–Huxley type have been used to explore electrical behavior and neurophysiological characteristics in neurons from several regions of the brainstem [30–35]. For example, some well-known phenomena associated with the neuronal electrical behavior such as spike frequency adaptation (SFA), post-stimulation recovery or delayed excitation, and postinhibitory rebound (PIR) have been related to specific ionic current mechanisms in medullary neurons [31,32] and in some cases these phenomena have been used to classify neurons by their electrophysiological behavior in response to electrical stimuli [30]. However none of these models examined specific electrical behaviors and related ionic mechanisms in individual neurons from chemosensitive regions, neither they have explored their intrinsic properties associated with their response to hypercapnic or acidotic stimuli.

The chemosensitive response of these neurons to acidotic stimuli affects respiratory, arousal, emotional and memory circuits. It is therefore important to study the responses of individual neurons to changes in pH and CO_2_, and to understand the cellular signaling mechanisms that govern autonomic and respiratory responses to such changes. This will, in turn, contribute to our understanding of the etiology of disorders with respiratory manifestations such as sudden infant death syndrome [12], sleep apnea [36], panic attacks [37–40], or Rett syndrome [41].

Given the potential wide-ranging impact of understanding the cellular mechanisms governing CO_2_/H^+^ sensitivity in neurons from chemosensitive regions, it is important to develop a model that faithfully replicates the effect of CO_2_/H^+^ on the activity of individual chemoreceptors. In the current study we propose a single cell model framework for a CO_2_/H^+^-sensitive neuron that mimics the general electrophysiological discharge patterns of locus coeruleus neurons at rest, as well as in response to electrical and chemical stimuli. Our main goal in this study was to investigate 1) intrinsic neuronal activity in absence of all possible external input from electrical and chemical stimuli; 2) the effects of excitatory and inhibitory stimuli on neuronal activity; and 3) the role that individual signals and chemical stimuli may play in the chemosensitive response.

## Results

In general, the model yielded regular activity at slow frequencies with the application of a small depolarizing current of 10–80 pA within a wide range of parameters. The method applied to integrate the system is also robust in the sense of converging to a bifurcation from resting activity to spiking over a wide range of starting parameter values.

For the set of parameter values used in this study, both electrical and some chemical signals play the role of bifurcation parameters. In particular, the equilibrium of the system loses stability with relatively high hyperpolarizing stimuli, be it an applied current of magnitude higher than 0.002 nA, an increase of the intracellular pH to higher values than 7.5, or an extracellular pH exceeding 7.58. Thus, spiking at arbitrarily low frequencies is only possible within a smaller range of parameters relative to the wide range at which higher frequencies are possible. This change in the state of the system from spiking to resting activity comes presumably from the ability of these parameters to move the dynamical system away from an Andronov-Hopf bifurcation, which prevents the model to oscillate at arbitrarily low frequencies.

### Validation of the model

The model was used to examine the typical firing properties of individual neurons that are common among various chemosensitive regions of the brainstem [42,43]. To assess intrinsic activity in absence of all possible input from electrical and chemical stimuli, the stimulus current (I_s_) was initially set to zero and CO_2_/H^+^ levels established at normocapnic conditions. Simulations resulted in spontaneous regular pacemaker-like activity characteristic of chemosensory neurons [44–48]. Some other characteristics, such as membrane properties and action potential shapes, were also within the range observed for these chemosensitive neurons at normal and hypercapnic conditions (Table 1).

**Table 1:** Experimental and calculated values for electrophysiological properties.

	Basic characteristics of action potential			Average chemosensitive response	
	Thresh.(mV)	AHP (mV)	Amplitude (mV)	Initial FR (Hz)	% Change in FR (HA)
**Exp.**	-48.5 ± 6.4	17.5 ± 2.5	76.3 ± 5.7	1.75 ± 1.25	119.5
**Model**	-50.3 ± 4.6	16.7 ± 2.2	70 ± 4.5	1.43 ± 2.19	99.3

Average values obtained with the neuron model in accordance with experimental data (Exp.) from spontaneous action potentials of LC chemosensitive neurons. Threshold, afterhyperpolarization (AHP), amplitude, initial firing rate (FR) and the percentage increase in FR at hypercapnic acidosis (HA). Experimental values were averaged with those taken from [18,29,43].

**Fig 1:**
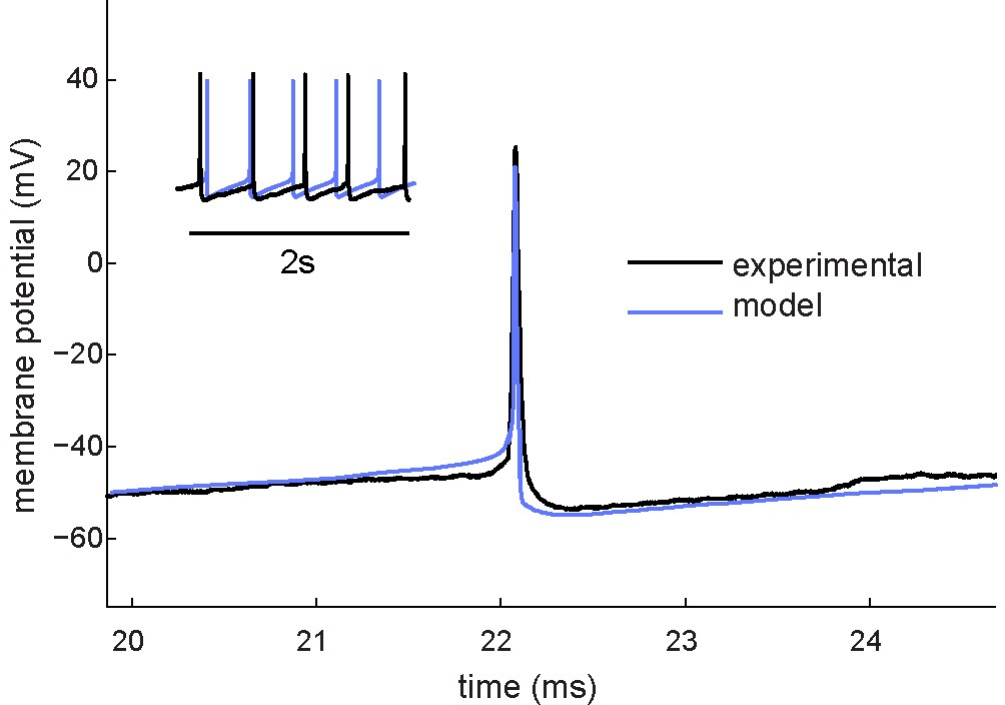
Comparison of experimental and model-generated data. LC neuron (black) and model-generated action potential waveforms (blue) were recorded during normal spontaneous activity. The model is capable of reproducing a similar pattern of AHP existent throughout the entire interspike interval observed in LC neurons.

In particular, simulations were run to predict some of the more important firing properties of individual chemosensitive neurons from the LC and to validate the model with intracellular recordings. As can be seen in Fig 1 the model results in spontaneous action potentials with a regular spiking pattern and with membrane properties (action potential shape, AHP phase, and frequency) similar to those observed in LC neurons.

### Neurocomputational aspects

To simulate the basic electrochemical behavior of a chemosensitive neuron, we subjected the model to electrical (from external stimulus current) and chemical (changes in CO_2_/H^+^) stimuli. Neuronal responses were distinguished by specific firing pattern phenomena, namely spike frequency adaptation, postinhibitory rebound, and post-stimulation recovery.

#### Spike frequency adaptation

**Fig 2:**
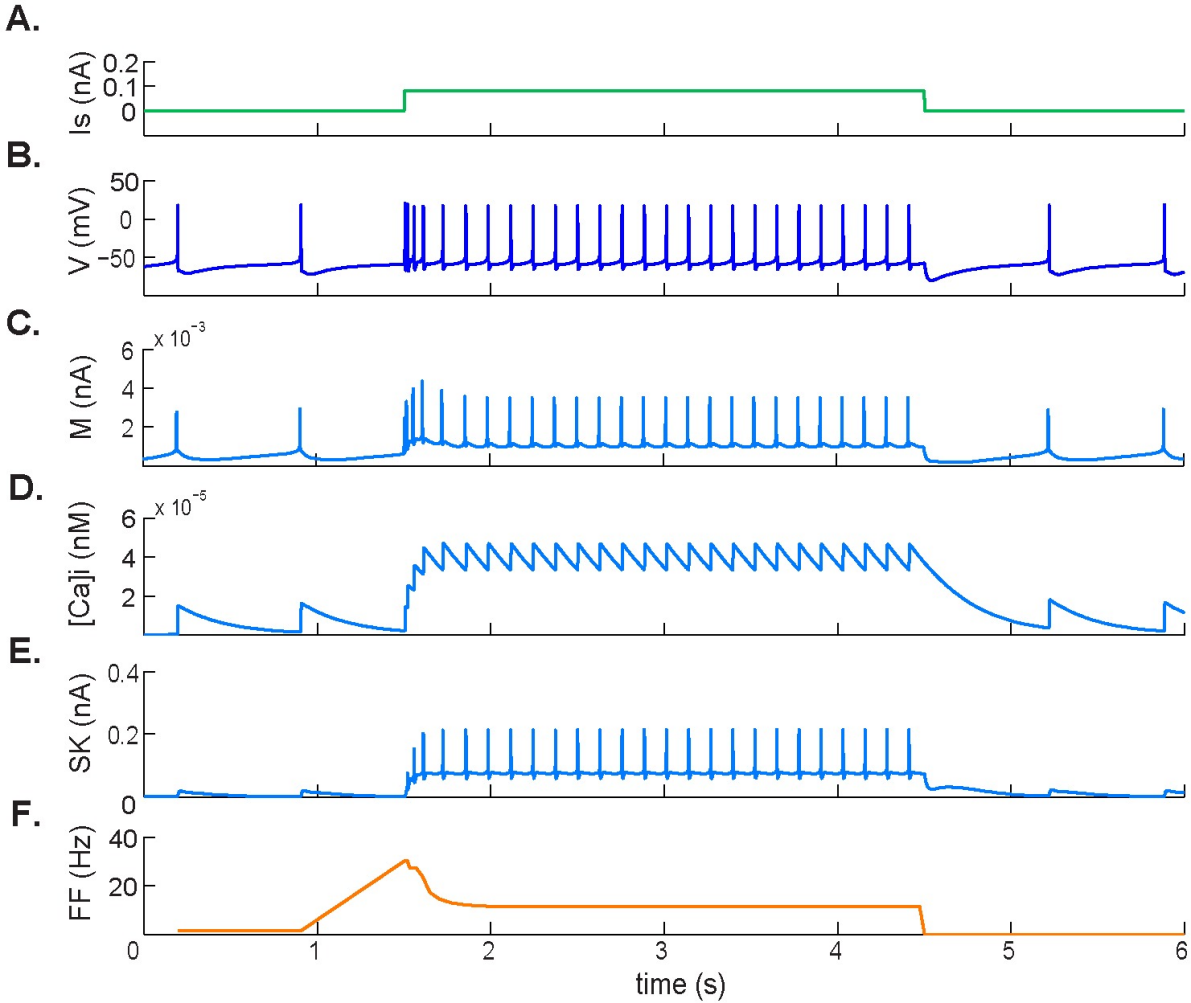
Spike frequency adaptation response to applied depolarizing current. **A.** Applied 0.1 nA depolarizing pulse. **B.** Simulated membrane potential during the pulse. **C.** M-type potassium current dynamics showing an increase in response to initial depolarization followed by a new, elevated steady state during stimulation. **D.** Intracellular calcium concentration [Ca^2+^]_i_. **E.** Calcium-activated SK-type potassium current. [Ca^2+^]_i_ and SK-type potassium current are both involved in the change in firing rate during depolarizing stimuli. **F.** Firing frequency.

The model-generated membrane potential during the application of a depolarizing current pulse is shown in Fig 2. In agreement with [29], depolarization within the range between 0.06nA and 0.21nA caused an initial increase in the firing rate, which adapted to a lower steady-state level. In particular, when applying a depolarizing current of 0.1 nA in normocapnia, we obtained a peak frequency of 30 Hz that adapted to a steady-state value of 8 Hz, resulting in an SFA ratio of 3.75 (Fig 2F).

**Fig 3:**
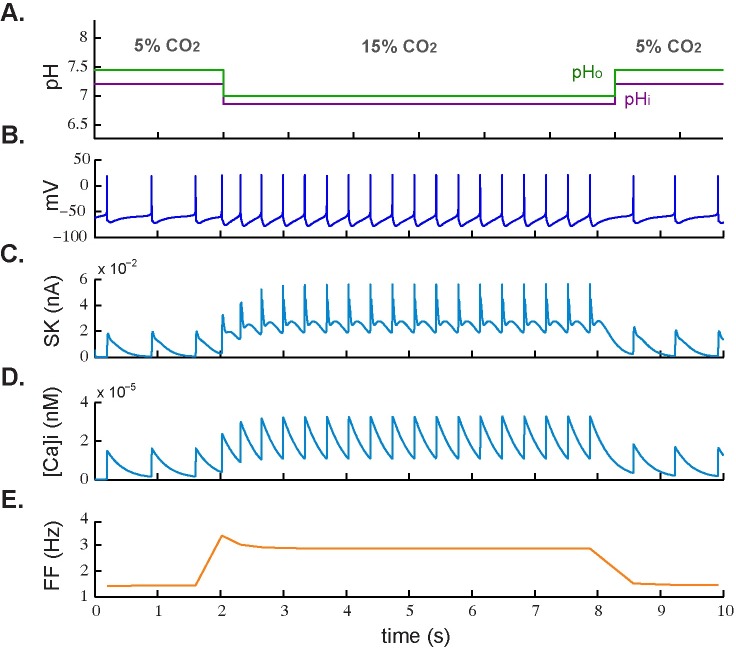
Spike frequency adaptation response to excitatory chemical stimulus. **A.** Simulated application of normocapnic (5% CO_2_) and hypercapnic acidotic (15% CO_2_) stimuli. **B.** Corresponding simulated membrane potential. **C.** Dynamic response of calcium-activated SK-type potassium current. **D.** Dynamic response of intracellular calcium concentration ([Ca^2+^]_i_). **E.** Firing frequency during CO_2_ level change.

Fig 3 shows the cell subjected only to an excitatory chemical stimulus. In Fig 3A and 3B, the simulated membrane potential is shown during an increase in CO_2_ level from 5% to 15% (hypercapnic acidosis) and the firing frequency adaptation can be seen in Fig 3E. With relatively low values for peak and steady-state frequency (3.5 Hz and 2.8 Hz, respectively), it resulted in an SFA ratio of 1.25.

#### Postinhibitory rebound

**Fig 4:**
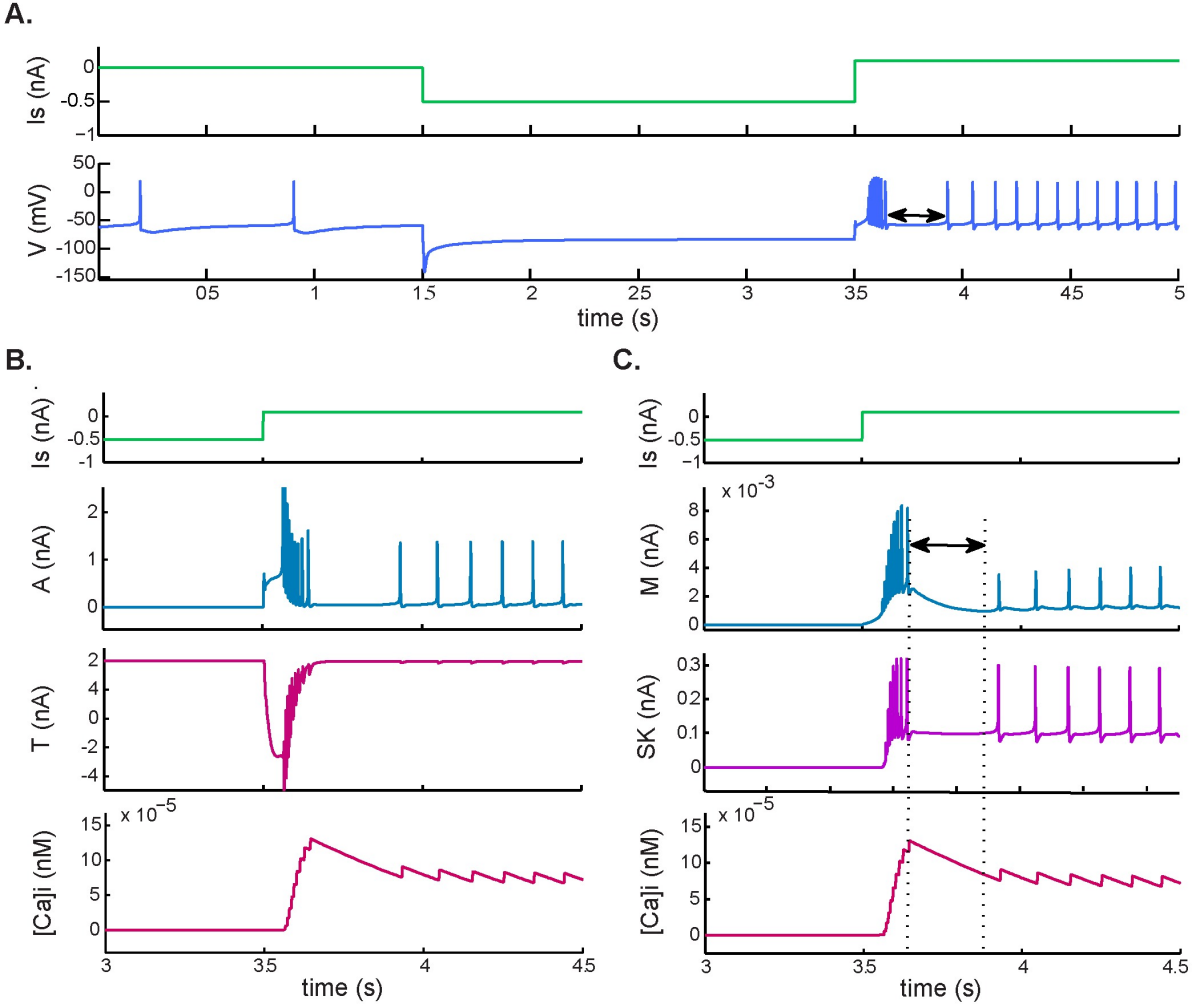
Postinhibitory rebound and post-stimulation recovery. Response to a hyperpolarizing pulse of −0.5 nA followed by a depolarizing pulse of 0.6 nA. **A.** Simulated membrane potential during current application. **B.** Postinhibitory rebound, involving an increase in A-type potassium and T-type calcium currents and an increase in intracellular calcium ([Ca^2+^]_i_). **C.** Post-stimulation recovery after postinhibitory rebound. This behavior was associated with accumulation of [Ca^2+^]_i_ and increased M-type and SK potassium currents.

Although this behavior has not been fully reported in LC neurons as far as we know, simulations resulted in spiking following a release from hyperpolarization, typical of some respiratory related neurons. Fig 4 shows the dynamic response of the membrane potential and the currents that were most affected when an inhibitory stimulus was released and followed by the application of a depolarizing current. This resulted in an increase in T-type current, which depolarized the membrane and promoted accumulation of intracellular calcium. There was also an increase in A-type current (Fig 4B) that limited the rebound spiking frequency.

#### Post-stimulation recovery

This passive electrophysiological property is characteristic of LC neurons and has been observed upon termination of hyperpolarizing currents around 0.5nA immediately followed by a depolarizing current of 0.2nA [29]. Simulations at the above range of hyperpolarizing and depolarizing currents and the resulting behavior is illustrated in Fig 4. Fig 4C shows the restoration of baseline membrane potential after the rebound that appeared due to the depolarizing pulse. Here, the electrical stimulus caused an increase in the average level of [Ca^2+^]_i_ and, upon termination of the rebound, the level of [Ca^2+^]_i_ started to decay. Once resting levels of [Ca^2+^]_i_ were restored, spiking resumed at an increased steady-state rate.

### Chemosensitive behavior

**Fig 5:**
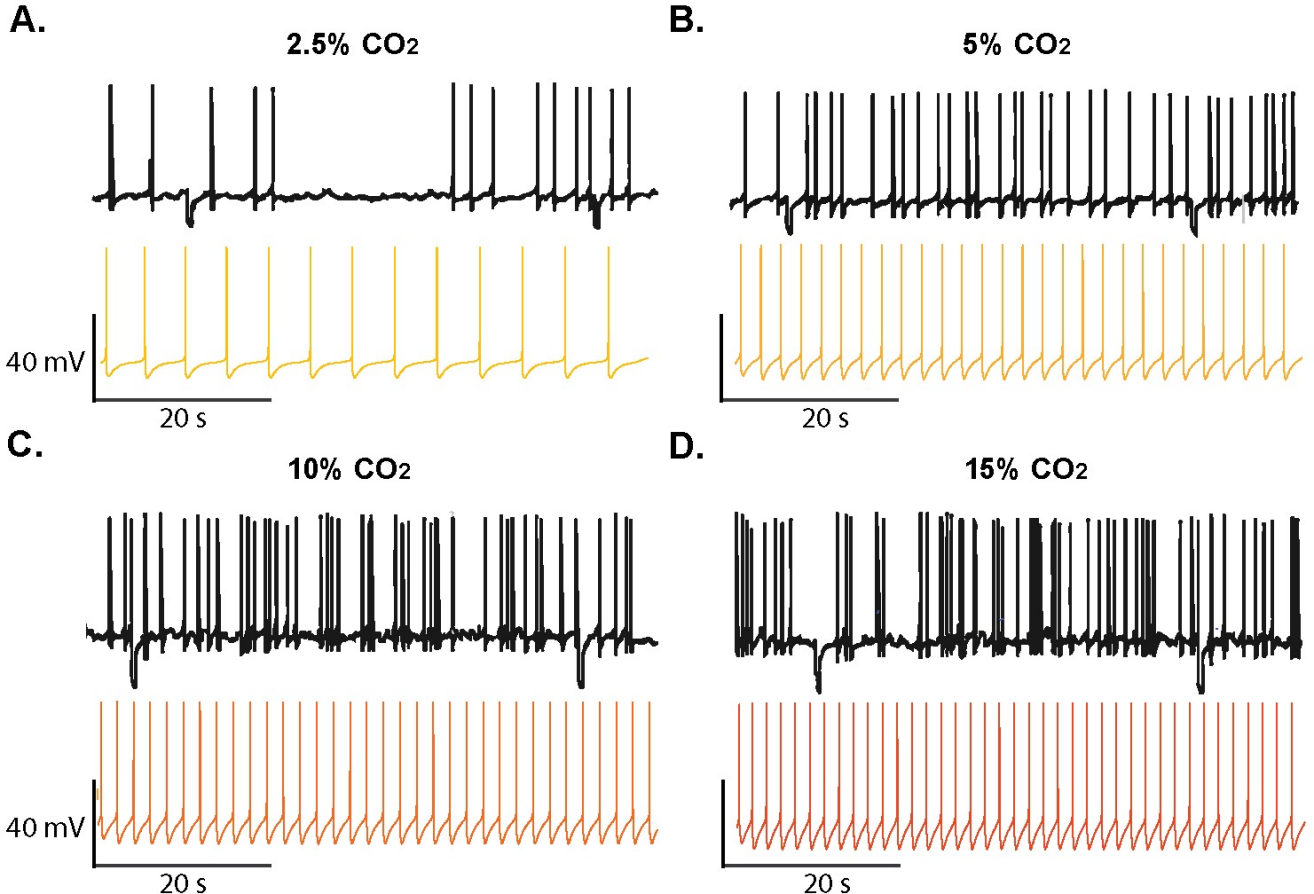
Comparison of simulated chemosensitive behavior and intracellular recordings. Effect of hypocapnia and hypercapnia on the spontaneous firing rate in locus coeruleus (LC) neurons from rats. **A.** Hypocapnic alkalosis (2.5% CO2, pH_o_ = 7.55, pH_i_ = 7.3). **B.** Normocapnia (5% CO2, pH_o_ = 7.45, pH_i_ = 7.23); **C.** Hypercapnia (10% CO2, pH_o_ = 7.15, pH_i_ = 7.4); **D**. Hypercapnic acidosis (15%CO2, pH_o_ = 7, pH_i_ = 6.96). Traces of membrane potential (Vm) vs. time showing action potential frequency from experimental data (black) and model-generated data (colored) for different conditions. Experimental traces were redrawn from data from Li and Putnam (2013) [45].

To simulate the basic chemosensitive behavior of a locus coeruleus neuron, we subjected the model to different levels of CO_2_/H^+^. As expected, neuronal responses were characterized by increased firing frequencies when related parameters where established to simulate hypercapnic and acidotic conditions in accordance with previous experiments in LC [49]. As illustrated in Fig 5 the predicted changes in frequency resulting from the chemosensitive response in the model are in agreement with intracellular recordings of LC neurons.

**Fig 6:**
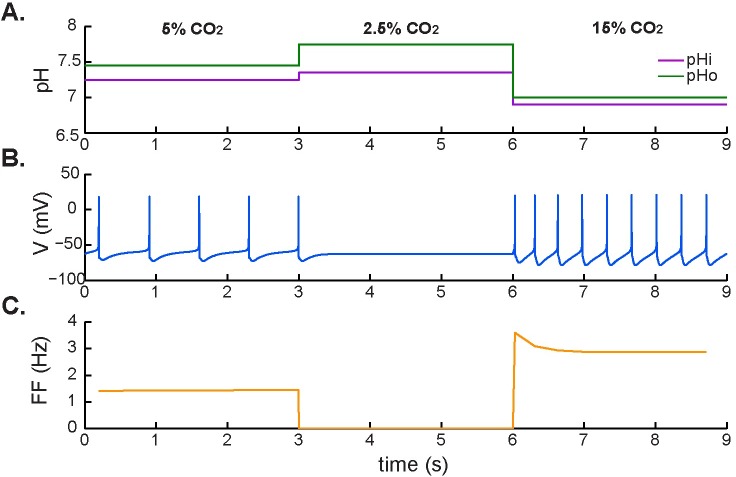
Neuronal response to hypocapnic and hypercapnic stimulation. **A.** Application of simulated normocapnia (5% CO_2_), hypocapnic alkalosis (2.5% CO_2_), and hypercapnic acidosis (15% CO_2_). **B.** Corresponding membrane potential. **C.** Firing frequency during changing chemical conditions.

We next investigated the effects of excitatory and inhibitory chemical stimuli (i.e. hypercapnic acidosis followed by hypocapnic alkalosis) on the tonic firing rate (Fig 6). In contrast with the electrical behavior in response to depolarizing and hyperpolarizing external stimuli, there was no evidence of postinhibitory rebound or post-stimulation recovery, and no depolarizing sag, when the stimulation was based on physiological CO_2_/H^+^ changes.

Here, the neuronal response to inhibition by hypocapnic acidosis (2.5% CO_2_) resulted in a hyperpolarization of the membrane potential that prevented the neuron from firing. This hyperpolarizing effect was released by subsequent hypercapnic acidosis (15% CO_2_), which made the neuron fire at higher frequencies and caused a new, elevated tonic firing rate compared with normocapnic conditions (5% CO_2_). There was no evidence for postinhibitory rebound before hypocapnic inhibition, consistent with a lower accumulation of [Ca^2+^]_i_ and inhibition of the L-type current during hypocapnic conditions.

### Effect of individual signals and chemical stimuli

To understand the effect of individual signals, whether electrical or chemical, on the activity of the neuron, we calculated the sensitivity of the firing-rate response to each signal (Table 2). A sensitivity index (SI) was thus defined as the percentage of control firing rate to which the activity increases or decreases in response to a change in each signal (Δs):

$$SI=\frac{\left|{FR}_{s}-{FR}_{c}\right|}{{FR}_{c}}∙100$$ (1)

where FR_s_ is the firing rate in response to a specific change, FR_c_ is the firing rate of the neuron in control (normocapnic) conditions (1.43 Hz), and Δs is the minimum increase or decrease of the signal necessary to produce an observable change in the firing rate. In other words, this value represents how much the neuron has to be stimulated with a particular signal in order to change its firing rate response from normal to altered activity. That is to say that in the case of chemical signals, a decrease in extracellular pH from 7.44 to 7.34, or an increase in the CO_2_ level from 5% to 7%, is enough to produce an increased firing rate response (see Table 2).

**Table 2:** Sensitivity of firing frequency to independent signals.

Signal	Control value	Δs	FR_s_ (Hz)	Slope (Hz/[s])	SI (%)
I_s_	0	0.02 nA	2.87	−71.9	100.7
CO_2_	5%	2%	1.46	0.014	2.09
pH_i_	7.25	0.1	1.78	−3.5	24.47
pH_o_	7.45	0.1	2.02	−5.9	41.26

Sensitivity (SI) relative to signal, calculated for different signals. As in Eq (1), it is defined as the percentage of control firing rate (FR_c_ = 1.43 Hz) to which firing rate increases or decreases in response to a change, Δs, in each signal. Signals: stimulus current (I_s_), carbon dioxide level (CO_2_), intracellular pH (pH_i_), extracellular pH (pH_o_).

**Fig 7:**
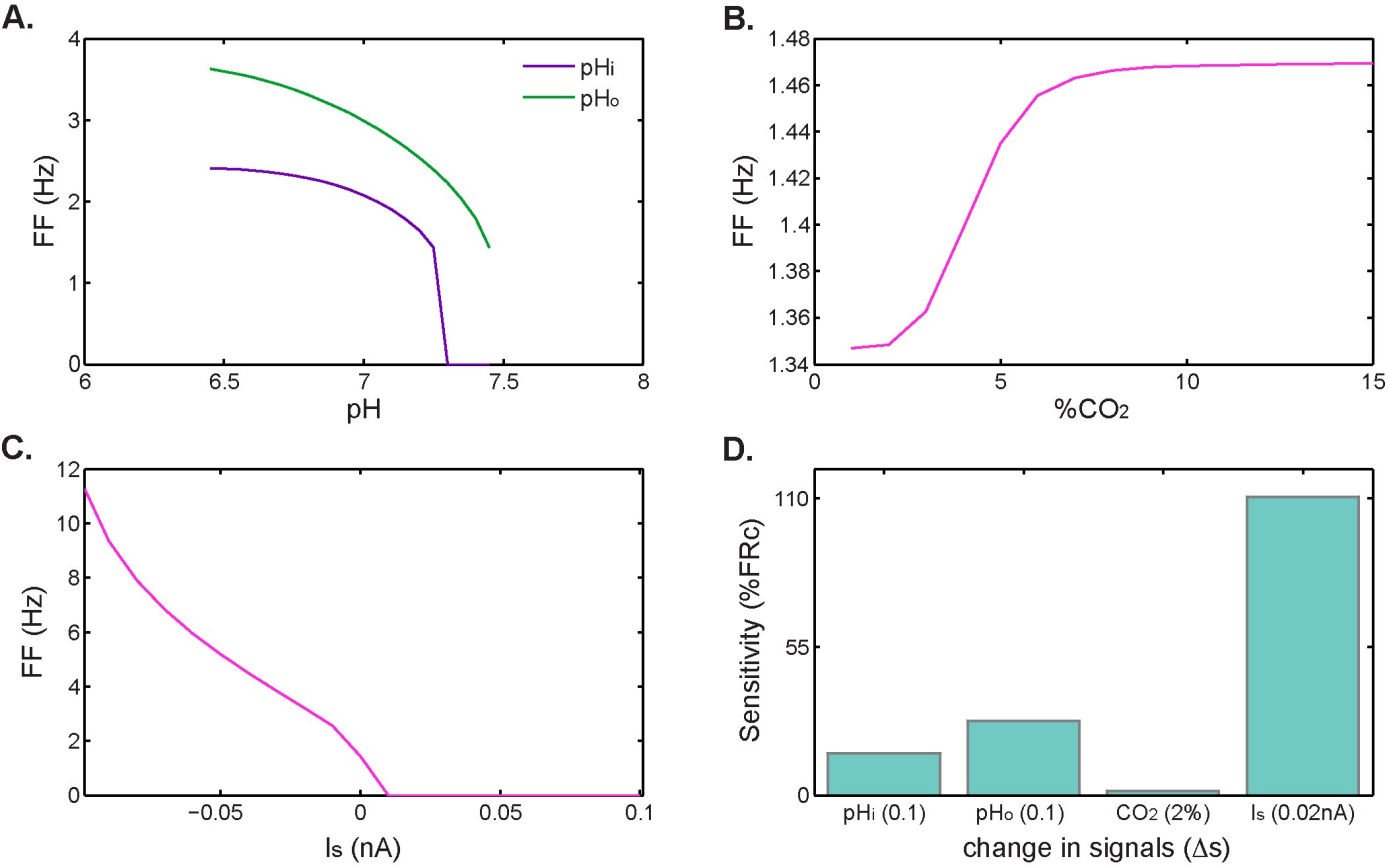
Effect of electrical and chemical signals on firing rate. **A.** Firing frequencies at different extracellular and intracellular pH levels, **B.** Firing frequency for different levels of CO_2_. **C.** Firing frequency for different external stimulus current values (negative values for depolarizing current. **D.** Contribution of each signal to the firing-rate response measured as the percentage change from control firing rate upon exposure to each signal.

Overall, the effect of applied stimulus current on the increased firing-rate response exceeds the contribution of chemical signals. According to calculated SI values, a small change in applied current (0.02 nA) can double the firing frequency, while a small reduction in pH (0.1 units) increases the firing rate by less than 42%. Small changes in %CO_2_ constitute the signal for which the model results are less sensitive, causing an increase in firing rate of about 2%. This effect is schematized in Fig 7. Here, the firing-rate response increases indefinitely with injected depolarizing current (Fig 7C), but saturates for chemical stimuli (Fig 7A and 7B).

**Fig 8:**
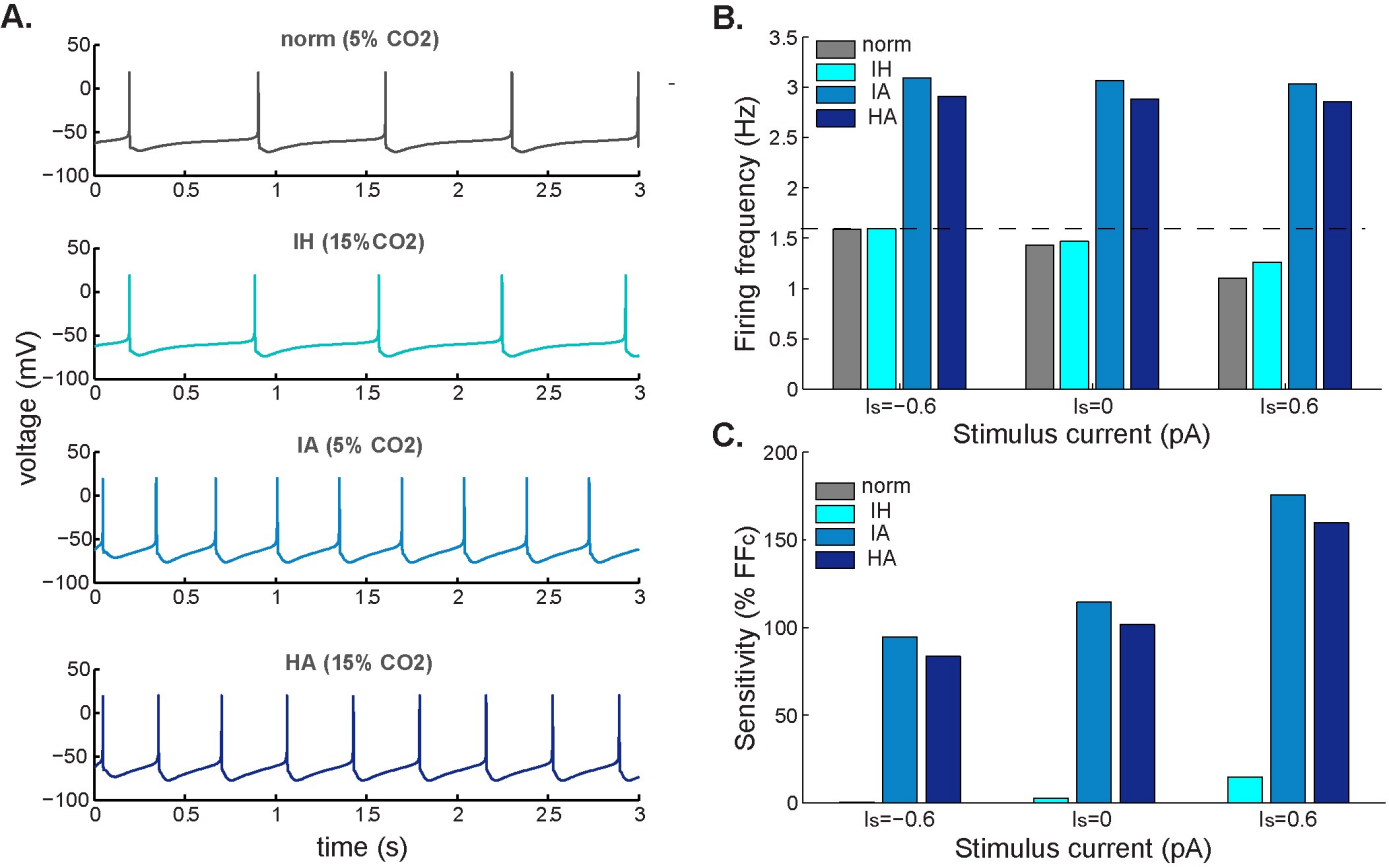
Firing frequency response to different electrical and chemical stimuli. **A.** Simulated membrane potential for different chemical stimuli: control (normocapnic: 5% CO_2_, pH_o_ = 7.45, pH_i_ = 7.25), isohydric hypercapnia (IH: 15% CO_2_, pH_o_ = 7.45, pH_i_ = 7.25), isocapnic acidosis (IA: 5% CO_2_, pH_o_ = 7.1, pH_i_ = 7.0), and hypercapnic acidosis (HA: 15% CO_2_, pH_o_ = 7.1, pH_i_ = 7.0). **B.** Firing rate under normocapnia (grey bar) and the same chemical stimuli as in A (blue bars) at different values of external applied current: excitatory stimulus current, I_s_ < 0; spontaneously firing neuron, I_s_ = 0; and inhibitory stimulus current, I_s_ > 0. **C.** Sensitivity for each chemical stimulus was calculated as the percentage increase from control firing rate.

Fig 8 shows the effect of external stimuli on the simulated membrane potential of a spontaneously active neuron in response to different chemical stimuli and the firing frequency for a set of conditions (normocapnia, isohydric hypercapnia, isocapnic acidosis, or hypercapnic acidosis) at different values of the stimulus current. We observed the combined effect of the applied current and the chemical signals for three different stimulating external currents (I_s_): depolarizing (I_s_ < 0), no electrical stimulus (I_s_ = 0), and hyperpolarizing (I_s_ > 0). From Fig 8B we noticed that whereas the firing frequency remained unchanged for acidotic conditions (i.e. IA and HA dark blue bars), it increased with excitatory input (I_s_ = -0.6) and decreased with inhibitory input (I_s_ = 0.6) at isohydric conditions (i.e. grey and light blue bars).

In addition, for each of the three acidotic conditions, we calculated the sensitivity of the firing rate at different stimulus currents using Eq 1 (see Table 3). Bars of the same color in Fig 8C represent the sensitivity of the firing-rate response to the same chemical condition but at different stimulus currents. In general, Fig 8C shows that the sensitivity of the model in each case increases with inhibitory input, as a consequence of an increase in the difference in firing rate between control (dark grey) and acidotic/hypercapnic conditions (blue bars), as can be also observed from Fig 8B.

**Table 3:** Sensitivity of firing frequency to different chemical stimuli.

Stimulus	pH_o_	pH_i_	%CO_2_	FR_s_	SI (%)
N	7.45	7.25	5	1.43	0
IH	7.45	7.25	15	1.26	11.9
IA	7.10	7.00	5	3.03	111.9
HA	7.10	7.00	15	2.85	99.3
HA*	7.10	7.25	15	2.68	87.4

The sensitivity (SI) is relative to the stimulus and is calculated for different chemical conditions. As in Eq (1), it is defined as the percentage of control firing rate (FR_c_ = 1.43 Hz) to which the firing rate changes (FR_s_) in response to a specific chemical stimulus. Stimuli: normocapnia (N), isohydric hypercapnia (IH), isocapnic acidosis (IA), hypercapnic acidosis (HA), hypercapnic acidosis with no change in intracellular pH (HA*).

## Discussion

We have developed a computational model of a spontaneously active single-neuron composed of CO_2_/H^+^-sensitive currents that reproduces the typical electrochemical behavior of individual brainstem neurons. The neuronal behaviors it describes have been observed both in computational models of neurons from different medullary regions and in experimental studies from brainstem chemosensitive nuclei [11,22,25,27]. The model specifically reproduces the regular pacemaker-like spiking pattern, action potential shape, and most of the membrane properties that characterize CO_2_/H^+^-sensitive neurons of the locus coeruleus.

### Neurocomputational aspects

Modulation of spike frequency is a fundamental intrinsic property regulating repetitive firing dynamics in response to constant stimuli. Our results demonstrate that during the stimulation, in response to an external current or an excitatory chemical stimulus, there is an adaptation of the firing rate that regulates the initial activity in response to depolarization (Figs 1 and 2). Results from previous studies in chemosensitive brainstem regions suggest that such modulation of the action potential frequency is determined by the amplitude of the afterhyperpolarization phase of the action potential, which can be triggered by the inactivation of Ca^2+^-activated K^+^ currents [29,31].

In agreement with previous studies, our results suggest that the mechanism of decay of the firing frequency responsible for SFA (Figs 2 and 3) and post-stimulation rebound (Fig 4C) involves an increase in calcium ion concentration inside the cell during depolarization. This accumulation of Ca^2+^ strengthens a calcium-activated K^+^ current (in this case the SK current) that transiently diminishes the initial firing rate (Fig 2D and 2E). The same mechanism was observed upon termination of the rebound in Fig 4C, where the level of [Ca^2+^]_i_ gradually started to decay. In both cases, once resting levels of [Ca^2+^]_i_ had been restored, spiking resumed at an increased steady-state rate.

In addition, we found that the modulation of the firing frequency during electrical stimulation was not only associated with intracellular calcium accumulation and corresponding SK current activation (Fig 2C and 2D), but also with an inhibitory effect of the M-type K^+^ current (Fig 2A–2C). In fact, there was less adaptation during chemical stimulation (reflected by a lower SFA value) where the M-type K^+^ current played no role. From these results, we propose that the M-type current plays a major role in modulating a high peak frequency during electrical stimulation and that the calcium-activated mechanism responsible for adaptation plays a more important role during chemical stimulation.

Previous studies on respiratory neurons suggest that the T-type calcium current is the main current responsible for the initiation and evolution of the postinhibitory rebound that results when the neuron is released from the hyperpolarizing stimulus followed by stimulation with a depolarizing pulse. In agreement with Rybak [32], we found that the dynamic response of the membrane potential under these conditions is associated with an increase in T-type Ca^2+^ current, which depolarizes the membrane and promotes accumulation of intracellular calcium. There is also an increase in A-type current that may limit the rebound spiking frequency (Fig 4B). In the same way, T-type current activation is associated with the initial depolarization that causes the rebound of action potentials and modulates its frequency as it decreases to its initial state.

During external electrical stimulation we also observed delayed excitation after the rebound (Fig 4C) due to a weaker activation of the SK current. In contrast with this response to depolarizing and hyperpolarizing external stimuli, there was no evidence of delayed excitation–not even a depolarizing sag was seen–when stimulation was based only on physiological CO_2_/H^+^ changes (Fig 6). This is in accordance with findings from a model of central CO_2_ chemosensitivity in *Helix aspersa* [44], which postulates that the A-type K+ current is the primary sensor for chemosensitive response to hypercapnic acidosis. However, we found that it is the M-type, not the A-type, K+ current that is most involved initiating the increased firing-rate response when stimulated with external input. In any case, we have no evidence from the present study that the M-type or pH-sensitive A-type potassium currents initiate the chemosensitive response to hypercapnic excitation, although Li & Putnam (2013) show a major role for A-currents in the hypercapnic response of locus coeruleus neurons [45].

### Chemosensitive behavior

There are two interesting findings from the model. First, the increased firing-rate response to excitatory stimuli seems to saturate at increasing levels of CO_2_ and/or acidification. This is in accordance with previous observations from the locus coeruleus, where a saturation effect seems to govern the neuron’s chemosensitive behavior [46]. Second, a higher chemosensitivity is obtained with this model when the chemical stimulus involves decreased intracellular and extracellular pH but not an increase in CO_2_ level alone. It can be seen from the relatively high value of the SI calculated for the increased firing rate in isocapnic acidosis. This observation is also strengthened by the relatively low value of SI that was obtained in isohydric hypercapnia, when the stimulus involved an increase in CO_2_ but the pH remained at the control level.

The fact that extracellular and not intracellular pH plays the major role in chemical signaling (Fig 7D) disagrees with a study by Filosa et al. (2002), in which the increased firing rate of locus coeruleus neurons in response to acid challenges correlated most with the magnitude and the rate of fall in pH_i_ [47]. This discrepancy can be explained in part because of the indirect limiting effect that pH_o_ has in the model. In other words, pH_o_ has the potential to inhibit the L-type calcium current, thus preventing the activation of the calcium-activated braking pathway and ultimately increasing the firing-rate response to acidotic challenges.

Indeed, the indirect effect that chemical signals may exert on the model’s response to hypercapnic acidosis constitutes an important limitation of the model. We assumed an additive effect of signals on membrane protein activity, but it is clear that there is an intricate interaction between activated and inhibited currents. For example, acid inhibition of a K^+^ channel could depolarize the membrane, which itself may activate another K^+^ current, thus reducing the initial depolarizing effect of the pH-sensitive channel. This interaction, and many others, makes the precise combined effect of different signals difficult to predict. However, the assumption of separate and additive effects of signals on membrane protein activity is supported by the previous observation that contributions of each current to the overall effect of hypercapnic acidosis are approximately additive [48].

Importantly, the present work confirms that small external electrical signals play a greater role in determining the chemosensitive response to changes in CO_2_/H^+^ than previously thought. This implies that a neuron can be erroneously classified experimentally as a chemosensitive or non-chemosensitive cell if external electrical factors (e.g. input resistance or gap junctions) are not properly blocked. Furthermore, this observation supports the notion that synaptic transmission is very likely to modulate the responses of chemosensitive neurons [48], and highlights the importance of considering synaptic transmission when defining and understanding key concepts such as intrinsic chemosensitivity.

## Model

The ionic current model describing neuronal activity is based on the Hodgkin–Huxley formalism, with membrane potential V and dynamics described as

$$c\frac{dV}{dt}=\sum_{i}I_{i}+I_{s},\;i\in \{Na,K_{dr},K_{ir},K_{A},K_{M},H,{Ca}_{L},{Ca}_{N},{Ca}_{T},BK,SK,B\}$$ (2)

where *c* is the neuron membrane capacitance (nF), *I*_*s*_ is a stimulus current (nA), representing an external stimulus coming from different sources (i.e. chemical synapses, gap junctions, injected currents, etc.) and *I*_*i*_ (nA) is the current flowing through the membrane channel *i*.

To propose a minimal configuration of an LC chemosensitive neuron the model incorporates different kinds of ion channels that have been identified in this nucleus, some of them known to play an important role in the intrinsic chemosensitive response of individual neurons. This set of membrane ionic currents consists of: transient sodium (Na) [29], delayed and inward rectifier potassium (Kir, Kdr) [24,43,45,50], transient potassium (K_A_, K_M_) [45,49], low and high threshold calcium (Ca_L_, Ca_T_) [18,51], calcium-dependent potassium channels (K_BK_, K_SK_) [19,29], and hyperpolarization-activated cation current [52]. We also included a background current (B) that accounted for the activity of non-selective leak potassium channels [53], and Na^+^/Cl^−^ currents flowing throughout the membrane owing to the action of intracellular pH-regulating transporters [29].

For voltage-dependent channels, each flowing current is expressed as

$$I_{i}=g_{i}{(E}_{i}-V)$$ (3)

where *g*_*i*_ (μS) is the conductance of channel *i*, and E_*i*_ (mV) is its equilibrium reversal potential (Table 4). The conductance associated with each channel is represented as

$$g_{i}={\varphi_{i}\bar{g_{i}}m}_{i}^{\alpha_{i}}h_{i}^{\beta_{i}}$$ (4)

where $\bar{g_{i}}$ is the maximal conductance of the *i* channel with activation and inactivation variables *m*_*i*_ and *h*_*i*_, respectively (Table 5). *φ*_*i*_, 0 < *φ*_*i*_ < 1 is a scaling factor that modulates the magnitude of the currents to account for the CO_2_/H^+^ sensitivity of the channels.

**Table 4:** Cell properties and ionic equilibrium potentials.

Cell Properties			Nernst Potentials (mV)	
**V_r_**	[mV]	−60	**E_K_**	−93
**R_in_**	[MΩ]	241.5	**E_H_**	−45
**A**	[mm^2^]	4	**E_Ca_**	60
**c**	[nF]	0.04	**E_Cl_**	−93
**I_s_**	[nA]	0	**E_Na_**	45

These biophysical properties are from a typical rat raphe neuron and were taken from refs [42] and [51]. V_r_: resting membrane voltage; R_in_: input resistance; A: total area including soma and dendrites; c: capacitance (estimated from A, using the 1 μF/cm^2^ rule); E_i_: Nernst or equilibrium potentials for the ion species. The external stimulus current, I_s_, was set to 0 to account for intrinsic neuronal response (e.g. spontaneous activity without external stimuli).

**Table 5:** Maximal conductance for ionic currents.

Inward currents	Conductance (μS)	Outward currents	Conductance (μS)
Na	0.5940	K_dr_	0.0384
H	0.0180	K_M_	0.0005
Ca_T_	0.1126	K_A_	1.5
Ca_L_	0.0005	SK	0.0030
Ca_L_	0.0005	TASK	0.002

This set of values for maximal conductance yielded regular spontaneous activity (I_s_ = 0) and were adapted from ref [42]. Maximal conductance for the TASK potassium current was set at 0.002 μS, which is in the range reported in ref [53]. Abbreviations for currents as in Eq 2.

The general formulation for the kinetics of ionic channels is based on previous brainstem single-neuron models [15,36]. Here, expressions for H^+^/CO_2_ sensitivity were included to each channel to account for chemosensory function and the resulting expressions were later fitted to experimental data in LC. Given the variability among identified neurons from LC, parameter values were optimized by eye, integrating the model for the appropriate current to fit individual currents under conditions that simulated normocapnic or hypercapnic acidotic experiments.

### Activation and inactivation dynamics

The rates of activation and inactivation for voltage-sensitive channels are defined as first-order differential equations of the general form:

$$\frac{dx}{dt}=\frac{x^{\infty }-x}{\tau_{x}},\;x\in \{m_{i},h_{i}\}$$ (5)

with steady-state values *x*^∞^

$$x^{\infty }=\frac{1}{1+e^{-(V-V_{x})/k_{x}}},$$ (6)

where *V*_*x*_ and *k*_*x*_ are half-activation voltage and slope factor, respectively (Tables 6 and 7). For each activation/inactivation variable *x*, time constant is defined by:

$$\tau_{x}=\left\{ \begin{array}{ll} a_{x}+b_{x}e^{-{\left(\left(V-V_{\tau_{x}}\right)/k_{\tau_{x}}\right)}^{2}} & \;if\;x\in \{m_{Na},h_{Na},h_{T}\}\\ \;a_{x}+\frac{b_{x}}{\cosh\left(\left(V-V_{\tau_{x}}\right)/k_{\tau_{x}}\right)} & \;otherwise \end{array}\right.$$ (7)

**Table 6:** Activation dynamics variables.

Par./current	units	Na	K_dr_	K_M_	K_A_	Ca_T_	Ca_L_	Ca_N_	BK	I_H_
**α**		3	1	1	4	2	2	2	1	1
**V_m_**	(mV)	-34.77	-15	-30	-57	-54.15	-20	-10	-20	-80
**k_m_**		10.5	7	9	8.5	6.2	8.4	7	2	5
**a_m_**		0.05	1	100	0.37	0.7	0.5	1	2	900
b_m_		0.15	4	0	2	13.5	1.5	1.5	0	---
V_τm_	(mV)	-43	-20	---	-55	-76	-20	-15	---	-80

Activation parameters for each current: number of gates, **α**_i_, half-activation voltage, V_m_, activation slope factor, k_m_, and specific parameters for the time constant function **a**_m_, **b**_m_, **V**_τm_. These values were taken from ref [42]. Where no unit is specified, parameters are unit-less values. Abbreviations for currents are as in Eq 2.

**Table 7:** Inactivation dynamics variables.

Par./current	units	Na	K_dr_	K_M_	K_A_	Ca_T_	Ca_L_	Ca_N_	BK	I_H_
**V_h_**	(mV)	-50.3	-78	-81	-45	-45	-50.3	-78	-81	-45
**K_h_**		6.5	6	4	13.8	10	6.5	6	4	13.8
**a_h_**		0.5	19	28	200	1000	0.5	19	28	200
b_h_		7.5	45	300	0	0	7.5	45	300	0
V_τh_	(mV)	-43	-80	-81	---	---	-43	-80	-81	---

Inactivation parameter values for each current: half-inactivation voltage, V_h_, inactivation slope factor, k_h_, and specific parameters for inactivation time constant function **a**_h_, **b**_h_, **V**_τh_. These values were taken from ref [42]. Where no unit is specified, parameters are unit-less values. Abbreviations for currents as in Eq 2.

### CO_2_/H^+^ sensitivity

We used the Hodgkin–Huxley formalism as a basis to include the effect of CO_2_ and H^+^ on the neuronal response. We implemented neuronal chemosensitivity in this ionic current model by quantifying the total CO_2_/H^+^ sensitivity of each channel and considering the excitatory and inhibitory role that hypercapnic and/or acidotic stimuli would have on individual channels due to the combined effect of independent signals.

To approximate the total sensitivity of each channel, we assumed separate and additive effects of signals on membrane protein activity [22]. Thus, we expressed CO_2_/H^+^ sensitivity as a unitless function (*φ*_*i*_ > 0):

$$\varphi_{i}=1-\sum_{s}w_{i,s}\varphi_{i,s},\;s\;\epsilon \;\{{pH}_{o},{pH}_{i},{\mathrm{CO}}_{2}\}$$ (8)

where *φ*_*i*,*s*_ defines specific functions for pH-dependent inhibition/activation of the channel *i*, and *w*_*i*,*s*_ is the contribution of the signal, *s* (extracellular pH, intracellular pH and/or CO_2_), to the present level of chemosensitivity. Here, a negative *w*_*i*,*s*_ accounts for channel activation and a positive value for inhibition. For non-chemosensitive currents, the contribution of *w*_*i*,*s*_ is set to zero (see Table 8).

**Table 8:** Contribution of chemical signals to CO_2_/H^+^ sensitivity in each channel.

weight/current	K_A_	K_dr_	K_ir_	Ca_T_	Ca_L_	BK	K_Leak_
***w*_pHi_**	0.3	1	1	0	0	1	0.5
***w*_pHo_**	0.7	0	0	1	0.3	0	0.5
***w*_CO2_**	0	0	0	0	0.7	0	0

Channel-specific weights assigned to each signal. Intracellular pH, ***w***_**pHi,**_, extracellular pH, ***w***_**pH0**_, and CO_2_ level, ***w***_**CO2**_. For non-chemosensitive currents the weight is set to zero. These values were adjusted to fit percentage of activation/inactivation of specific currents in different acidotic challenges [23,45,47,49]. Abbreviations for currents as in Eq 2.

In accordance with [54], we assumed that pH sensitivity (due to changes in pH_o_ or pH_i_) relies on titrable amino acid residues in the channel protein. In the case of CO_2_-dependent activation, CO_2_ molecules are supposed to bind the channels and promote a second open state that enhances its activity [55]. As both situations describe a typical saturation effect, we express the relation between each signal and current inhibition/activation as a Hill equation:

$$\varphi_{i,s}(s)=\frac{1}{1+{\left(s_{1/2}/s\right)}^{h_{s}}}$$ (9)

where *s*_1/2_ corresponds either to the pK (pH level at the midpoint of current inhibition) or to the percentage of CO_2_ required to achieve a half-maximal activation (Table 9). Hill coefficients *h*_*s*_ for each curve represent the sensitivity to each stimulus. Parameter values for intracellular and extracellular pH responses (titration curves) are shown in Table 10.

**Table 9:** Signal values at midpoint current inhibition/or activation.

S_1/2_/current	K_A_	K_dr_	K_ir_	Ca_T_	Ca_L_	BK	K_Leak_
**pK_i_**	7.4	7.1	7	---	---	6.5	7.3
**pK_o_**	7.3	---	---	6.9	6.5	---	7.4
**(CO_2_)_½_**	---	---	---	---	5	---	---

Midpoint values for current inhibition/activation curves. A pK value was assigned to intracellular (pK_i_) and extracellular (pK_o_) pH titration curves. The percentage of CO_2_ required to achieve half-maximal current activation, (CO2)½ was calculated as an approximation to fit data from ref [19]. Abbreviations for currents as in Eq 2.

**Table 10:** Hill coefficients for titration/saturation curves.

Hill constant/current	K_A_	K_dr_	K_ir_	Ca_T_	Ca_L_	BK	K_leak_
*h_i_*	15	15	15	---	---	2	1
*h_o_*	15	0	---	1	1	---	---
*h_c_*	---	---	---	---	5	---	---

Hill coefficient values for current’s titration curve(s). Hill values in the table were calculated by fitting reported titration curves that allowed specific % change in ionic currents when different acidotic challenges were applied [23,45,47,49]. *h*_*i*_ for changing intracellular pH, *h*_*o*_ for changing extracellular pH, and *h*_*c*_ for changing CO_2_ level. Abbreviations for currents as in Eq 2.

### Calcium dynamics and Ca^2+^-activated currents

#### Calcium-activated currents

Following ref [42] we assumed no explicit inactivation process for SK currents as its decay depends only on [Ca^2+^]_i_. We used a calcium-dependent steady-state activation variable for the SK current:

$$m_{SK}^{\infty }=\frac{{Ca}_{in}^{n}}{{Ca}_{in}^{n}-K_{c}^{n}}$$ (10)

where *n* is the Hill coefficient and *K*_*c*_ is the value of *Ca*_*in*_ at half-activation for $m_{SK}^{\infty }$ (Table 11). A recent study of firing activity on raphe neurons provided a more accurate estimate of the big potassium channel (BK) current by using a simplified model, in which, instead of considering the Ca^2+^ dependence of the BK channel conductance, it was coupled with N-type Ca^2+^ [42]. As there is evidence that BK channels are linked to L-type voltage-gated calcium currents in the rat brain [56], we used the Ca^2+^ current-coupled BK model for this current. The BK conductance was therefore coupled to the L-type Ca^2+^ channel, so the current was expressed as

$$I_{BK}=g_{BK}L(E_{i}-V)$$ (11)

where *L* is a unitless value representing the magnitude of the L-type Ca^2+^ current.

**Table 11:** Calcium dynamics and SK parameter values.

Ca dynamics parameters			SK activation parameters		
***K_d_***	(mM)	0.001			
***CFS***	---	0.201	***Ca_in_***	(nM)	50
***F***	(Cmol^-1^)	96500	***n***	---	2
***d***	(μm^2^)	0.062	***K_c_***	(mM)	25
***v***	(mm^3^)	0.25	***α***	---	1
***K_s_***	(nMs^-1^)	0.390625	***a_m_***	(ms)	5
***K_m_***	(mM)	0.0001	***b_m_***	(ms)	0
***B_tot_***	(mM)	0.03			

These values were taken from [42]. The shell volume *v* was calculated as the total area of the neuron × shell thickness (Table 4). Abbreviations for Ca dynamics as in Eqs 12 and 13. Parameter values for BK activation: **α**_i_, is the number of activation gates, and **a**_m_, **b**_m_ are specific parameters for the time constant function (Eq 7). Other abbreviations for BK activation as in Eq 10.

#### Calcium ion dynamics

The dynamics of free Ca^2+^ are assumed to occur inside a thin shell volume *v*, determined by the thickness and total area of the cell (see Table 4). Free calcium ions outside the neuron flow into it through voltage-gated Ca^2+^ channels and are then buffered or pumped out of the neuron by membrane transporters like the plasma membrane Ca^2+^ ATPase pump. Thus, [Ca^2+^]_i_ dynamics can be described by the following differential equation:

$$\frac{d{Ca}_{in}}{dt}=\frac{-CSF(I_{Ca})}{2Fv}∙(1-P_{B})-K_{s}\frac{{Ca}_{in}}{{Ca}_{in}+K_{m}}$$ (12)

where *v* is the volume of a thin shell just inside the membrane and F is the Faraday constant, and *I*_*Ca*_ represents the sum of high-threshold calcium currents. We included a calcium source factor (CSF; 0 < CSF < 1) that, where necessary, regulates the extent to which the internal calcium ion concentration is due to the calcium ions flowing through the currents [42]. The last (Michaelis–Menten) expression in the above equation is used to describe the pump mechanisms that maintain a low [Ca^2+^]_i_, where *K*_*s*_ is the maximal pumping rate and *K*_*m*_ is the dissociation constant ([Ca^2+^]_i_ for half-maximal rate). The bound fraction of Ca^2+^, *P*_*B*_, is given by

$$P_{B}=\frac{B_{tot}}{{Ca}_{in}+B_{tot}+K_{d}}$$ (13)

with *B*_*tot*_ representing the sum of the free and bound buffer and *K*_*d*_ being the dissociation constant of the reversible reaction underlying the binding of buffer B to Ca^2+^. These models for BK channel and Ca^2+^ dynamics are described in [42] and the parameter values are listed in Table 11.

### Background currents

CO_2_/H^+^-dependent inward rectification has been observed in adult and neonatal rat neurons from various chemosensitive regions. We therefore included an inwardly rectifying current (I_Kir_), with a reversal potential of −44 mV taken from [31]:

$$I_{K_{ir}}={\bar{g}}_{K_{ir}}\frac{\left(V-E_{K}-36\right)}{1+exp\{(V-E_{K}+140)ZF/RT\}}$$ (14)

Assuming that there is a non-zero ion flux at rest, as the neuron fires spontaneously without afferent input, we added a small background current stated to be composed of chloride, sodium and potassium. Our reason for including these leak currents is because intracellular pH regulation by acid–base transporters results in altered neuronal excitability and their effect might be brought about by the pH modulation of Na^+^/H^+^ and Cl^−^/HCO^3−^ exchangers [57]. In addition, pH_o_-sensitive K^+^ leak currents like the TASK-1 channel are expressed in chemosensitive neurons [58] and seem to be involved in the chemosensitive response of these neurons to hypercapnia. As the resting membrane potential will also be affected by the action of these factors, this change can be modeled by the following approximate formula, which assumes that sodium and chloride ion flux, from pH_i_-regulating proteins, balances leak K^+^ flux:

$$I_{B}=\sum_{j}I_{j,leak}\;j\in \{Na,K,Cl\}$$ (15)

with different leak currents expressed as linear *I*_*j*,*leak*_ = *g*_*j*,*leak*_ (*V*–*E*_*j*_). As the total leak conductance can be estimated as the reciprocal of the neuron’s input resistance we obtain:

$$g_{K,leak}=g_{task}$$ (16)

$$g_{Cl,leak}=g_{leak}-g_{K,leak}-g_{Na,leak}$$ (17)

$$g_{Na,leak}=\frac{{\left(R_{in}\right)}^{-1}(E_{Cl}-V_{r})+g_{K,leak}(E_{Cl}-E_{K})}{E_{Cl}-E_{Na}}$$ (18)

where *V*_*r*_ is the membrane potential that gives an equivalent leak equilibrium potential around −65 mV [36] and *R*_*in*_ is the input resistance (Table 4).

The model was implemented in a MatLab programming environment using a variable-step differential equation solver. The resulting ODE system given by Eqs (2), (5) and (12) was solved numerically using the stiff variable-step differential equation solver ode15s. For each simulation, the model was run until steady-state conditions, from which data and calculations were then collected.
